# Concentration of soybean lecithin affects short-term storage success of goat semen related with seminal plasma removal

**DOI:** 10.21451/1984-3143-AR2019-0012

**Published:** 2019-11-18

**Authors:** Robespierre Augusto Joaquim Araújo Silva, André Mariano Batista, Lúcia Cristina Pereira Arruda, Helder Melo de Souza, Igor Henrique de Azevedo Valença Nery, Wilton Arruda Gomes, Pierre de Castro Soares, Sildivane Valcácia Silva, Maria Madalena Pessoa Guerra

**Affiliations:** 1 Universidade Federal Rural de Pernambuco Laboratório de Andrologia Departamento de Medicina Veterinária RecifePE Brasil Universidade Federal Rural de Pernambuco, Laboratório de Andrologia, Departamento de Medicina Veterinária, Recife, PE, Brasil; 2 Universidade Federal Rural de Pernambuco Laboratório de Doenças Metabólicas e Nutricionais de Ruminantes Departamento de Medicina Veterinária RecifePE Brasil Universidade Federal Rural de Pernambuco, Laboratório de Doenças Metabólicas e Nutricionais de Ruminantes, Departamento de Medicina Veterinária, Recife, PE, Brasil; 3 Universidade Federal da Paraíba Centro de Biotecnologia João PessoaPB Brasil Universidade Federal da Paraíba, Centro de Biotecnologia, João Pessoa, PB, Brasil

**Keywords:** buck sperm, phosphatidylcholine, liquid storage semen, phospholipase A2

## Abstract

The objective of this study was to investigate the need of seminal plasma removal for short-term cooling of buck semen in soybean lecithin (SL) based extender. Each pool was divided equally, and one half was subjected to centrifugation to remove seminal plasma (SP-), while the other half remained with seminal plasma (SP+). Then, both SP+ and SP- samples were diluted in two SL extenders (extender A = 1% SL; extender B = 2% SL), cooled to 5ºC and stored for 48 hours. The sperm kinetics, evaluated by CASA, and plasma membrane integrity (PMI), acrosomal integrity (ACI) and high mitochondrial membrane potential (HMMP), evaluated by epifluorescence microscopy, were determined within five minutes after reaching 5°C (T0), as well as after 24 (T24) and 48 (T48) hours of storage. Interactions (seminal plasma *vs.* extender *vs.* time;) were observed for all variables assessed. Total and progressive motility and other variables of sperm kinetics decreased after 24 hours of cooling in the SP+ group, and after 48 hours of storage, these same variables were lower in SP+/B compared to SP-/B groups. Furthermore, SP+ reduced PMI (extender B, T48), HMMP (A and B extenders, T48) and ACI (extender A, T0) compared to SP- samples. The interactions between seminal plasma and soybean lecithin phospholipids seemed to occur in a time-dependent manner. It was concluded that the removal of seminal plasma improves the quality of goat semen that was cooled in a soybean lecithin-based extender, especially when using 2% soybean lecithin.

## Introduction

Liquid-stored semen can be an alternative to frozen-thawed semen for artificial insemination, since semen cryopreservation is an expensive process ( Liu et al., 2016 ). Although much research has already been conducted to prolong the *in vitro* viability and fertilizing potential of stored liquid semen, limited improvements have been achieved in caprine species ( Paulenz et al., 2005 ; Xu et al., 2009 ; Zhao et al., 2009 ).

Traditional extenders for goat semen cryopreservation include egg yolk, skimmed milk or their combination ( Purdy, 2006 ). However, enzymes in the seminal plasma of bucks may interact with specific components of skimmed milk or egg yolk, rendering extenders containing these substances harmful to the bucks’ spermatozoa ( Pellicer-Rubio et al., 1997 ; Leboeuf et al., 2000 ; Aboagla and Terada, 2004 ). In addition, the hygienic risks and lack of quality standards associated with the use of animal products in cryoprotective media has stimulated the search for substitutes, preferably of non-animal origin, capable of conferring cryoprotection to male gamete as well as biosecurity ( Bousseau et al., 1998 ).

In the last decades, many researches have reported the replacement of the animal components with soybean lecithin (SL) for preservation of ovine, bovine and caprine semen ( Gil et al., 2003 ; Celeghini et al., 2008 ; Vidal et al., 2013 ; Salmani et al., 2014 ; Chelucci et al., 2015 ). The SL contains a mixture of phospholipids, fatty acids and low-density lipoproteins, which protects the sperm cell membranes by restoring the phospholipids lost during heat shock ( Forouzanfar et al., 2010 ; Oke et al., 2010 ).

The removal of seminal plasma by washing before semen dilution is often recommended to improve the quality of frozen-thawed goat semen when milk- or egg yolk-containing extenders are used ( Leboeuf et al., 2000 ; Purdy, 2006 ). Soy lecithin differs from egg yolk or skimmed milk in lipid composition and fatty acid content ( Küllenberg et al., 2012 ; Mutalik et al., 2014 ) and might interact differently with the enzymes in goat seminal plasma.

Data on the effects of the interaction between SL-based extenders and seminal plasma in regards to semen quality of buck sperm are scarce and contradictory ( Sariözkan et al., 2010 ; Roof et al., 2012 ). Of note, there are reports on frozen-thawed goat spermatozoa in commercially available extenders containing a substitute for egg yolk like Bioxcell ^®^ . To our knowledge, no study is available on the liquid preservation of goat semen in prepared extenders containing soya lecithin and its interaction with seminal plasma.

With this in mind, the objective of this study was to investigate the need to remove seminal plasma for short-term goat semen storage in two soybean lecithin-based extenders.

## Materials and methods

### Chemicals

Unless otherwise indicated, all reagents were purchased from Sigma Aldrich Co (St. Louis, MO, USA).

### Animals, semen collection and analysis

Four sexually mature Toggenburg bucks with fertility histories, individually housed at the Department of Veterinary Medicine of the Universidade Federal Rural de Pernambuco (UFRPE), Recife, Brazil (8°03’14” S and 34°52’52” W), were used in this study. They were fed 400 g/animal/day of a commercial concentrate and Tifton hay, water and mineral salts *ad libitum* . All procedures were approved by the Ethics Committee for Animal Use of the UFRPE under license number 008/2014 CEUA/UFRPE.

The ejaculates were obtained with the artificial vagina method, using a female as a dummy. Eight ejaculates were collected per buck (two collections per week), for a total of 32 ejaculates. After collection the ejaculates were analyzed subjectively and only when samples presented > 0.8 mL in volume and ≥ 60% motility, the ejaculates were pooled. Then, the pools were analyzed and those that presented ≥ 60% motility, ≥ 1 × 10 ^9^ spermatozoa/mL and ≤ 20% abnormal spermatozoa were approved for use in further experiments.

### Preparation of the extenders

Tris buffer (250.0 mM Tris, 88.5 mM citric acid, 69.38 mM glucose, 10.0 mM HEPES, 100 IU penicillin, 50 mg streptomycin, 100 mL ultrapure water; 320 mOsm and pH 7.2) was used as a base extender for production of extenders A and B, composed of 1 or 2% soybean lecithin, respectively.

To prepare soybean lecithin-based extender (SL), 1 or 2% (w/v) of soybean lecithin (P5638) were added to the base extender and kept hydrated at room temperature (25 °C) for 1 h. Afterward, the extenders were homogenized in a magnetic stirrer to form a homogeneous solution. Subsequently, the extenders were kept in an ultrasonic bath for 30 min (25 °C) for fragmentation of the micelles, following the methodology described by Mozafari (2010) . After sonication, debris were removed. The solutions were clarified by centrifugation (2,200 × g for 30 min) and then filtered twice: first through a 3 µm pore (Cellulose Ester Filter, SSWP04700, Merck-Millipore, USA) and then through a 0.45 µm pore (Chromafil® Xtra PVDF-45/25, Macherey-Nagel GmbH & Co., Germany), as described by De Paz et al. (2010) with modifications.

### Experimental procedure

Each pooled sample was split into two equal fractions, one of which contained seminal plasma (SP+) and other with the seminal plasma removed (SP-). Seminal plasma removal was performed according to Soares et al. (2015) . Briefly, pooled semen samples were diluted (1:9, v/v) in washing solution (297.59 mM Tris + 105.35 mM citric acid + 82.58 mM fructose + 100 mL ultrapure water; 330 mOsm and pH 6.8) and centrifuged twice at 1,750 × g for 10 min.

After the washing procedure, both fractions (SP+ and SP-) were diluted in A and B extenders (200 × 10 ^6^ sperm/mL), forming four experimental groups: SP+/A (with seminal plasma, extender A), SP+/B (with seminal plasma, extender B); SP-/A (without seminal plasma, extender A) and SP-/B (without seminal plasma, extender B). Diluted semen samples were then packaged into 0.25 mL plastic straws and cooled in an automated system (TK-3000®, TK Tecnologia em Congelação LTDA, Uberaba, Brazil) using a curve cooling (-0.25 °C/min to 5 °C). After reaching 5 °C (90 minutes), straws were transferred to a refrigerator (5 °C) and kept in a horizontal position for up to 48 hours.

### Semen evaluation

The evaluations were carried out at three different times: five minutes after reaching 5 °C (T0) and after 24 hours (T24) and 48 hours (T48) of storage. To perform the analysis, two straws of from treatment were warmed in a 37 °C water bath for 30 s ( Peterson et al., 2007 ) and was again diluted in its own extender (37 °C) to concentrations of 20 × 10 ^6^ sperm/mL. Then, aliquots of these samples were used to evaluate the sperm kinetics by computer-assisted semen analysis (CASA), plasma membrane integrity, acrosomal integrity and high mitochondrial membrane potential through epifluorescence microscopy (Carl Zeiss, Göttingen, Germany).

The sperm kinetics evaluation was performed using CASA (SCA ^TM^ ; Microptics, S.L., Version 5.1, Barcelona, Spain). A 5.0 µL aliquot of the sample was placed on a pre-warmed slide (37ºC), which was covered with a coverslip and evaluated by means of phase contrast microscopy (Eclipse 50i, Nikon, Japan); the images were captured using a video camera (Basler Vision Technologie ^TM^ A312FC, Ahrensburg, Germany). The sperm motility estimations were performed in five random and non-consecutive microscopic fields for each sample by the same operator. The following variables were evaluated: total motility (TM; %), progressive motility (PM; %), linearity (LIN; %), straightness (STR; %), wobble (WOB, %), curvilinear velocity (VCL; µm/s), straightline velocity (VSL; µm/s) and average path velocity (VAP; µm/s). The CASA was set up as follows: temperature 37 °C; magnification, 100x; number of frames, 25; images per second, 25; head area, 20 to 70 µm ^2^ ; VAP: slow 10 µ/s < middle 45 µ/s < fast 75 µ/s; progressiveness, 80% STR; circular, 50% LIN.

The plasma membrane integrity (PMI) was assessed through the double-staining method using the combination of carboxyfluorescein diacetate (CFDA; 0.46 mg/mL in DMSO) and propidium iodide (PI; 0.5 mg/mL in PBS). For each treatment, an aliquot of the sample (30 µL) was stained with 5.0 µL CFDA and 5.0 µL PI and incubated for 10 min (25 °C). Two hundred sperm were assessed using DBP 485/20 nm excitation and DBP 580–630 nm emission filters at a magnification of 400x. Sperm stained only in green were considered to have intact membranes and those stained red had damaged membrane.

The acrosomal integrity (ACI) was assessed using fluorescein isothiocyanate dye conjugated to *Peanut Agglutinin* (FITC-PNA; 100 µg/mL in PBS). For each treatment, a 10 µL aliquot of the sample was used to make a smear, which was air-dried, stained with 30 µL FITC-PNA and then incubated in a humidity chamber at 4 °C for 15 minutes in the dark. Then, the slides were immersed in PBS twice and air-dried naturally. Immediately before evaluation, 5.0 µL of the solution (4.5 mL glycerol, 0.5 mL PBS and 5.0 mg p-phenylenediamine) was placed on the slide and covered with a coverslip. Two hundred sperm were assessed using BP 450–490 nm excitation and LP 515 nm emission filters at a magnification of 400x. Sperm in which the acrosome region was stained fluorescent green had intact acrosome, and when only the equatorial region of the sperm head was fluorescent green or when fluorescence was absent, the sperm had a damaged acrosome.

The high mitochondrial membrane potential (HMMP) was assessed using a lipophilic cationic fluorochrome (JC-1; 0.15 mM in DMSO). For each treatment, a 30 µL aliquot of the sample was stained with 5.0 µL JC-1 and incubated for 10 min (25 °C). Two hundred sperm were assessed using BP 450–490 nm excitation and LP 515 nm emission filters at a magnification of 400x. Sperm with midpieces stained orange had a high mitochondrial membrane potential, while sperm with midpieces stained green had low mitochondrial membrane potential.

### Statistical analysis

Data were evaluated using IBM® SPSS® Statistics for Windows. First, data were tested for normality using the Kolmogorov-Smirnov test. Data with residuals not normally distributed (PMI, HMMP, ACI, TM, PM, LIN, STR, WOB) were transformed using the arcsine method (arcsine √P/100). Then, data were submitted to analysis of variance (F-test) using the GLM (General Linear Model) procedure with time-repeated measures. Analysis of variance (ANOVA) separated the effects of seminal plasma (SP), extender (E), cooling time (T) and interactions between these factors (SP x E, SP x T, E x T, SP x E x T) as causes of variation. When the F-test showed significant differences ( *p* < 0.05), the means were compared using the Bonferroni post-test ( *p* < 0.05). The results are expressed as the mean ± standard error of the untransformed data. This experiment was repeated eight times.

## Results

Significant interactions were observed between seminal plasma (SP), levels of soybean lecithin (E) and cooling time (T) for all variables analyzed in this study. Thus, the effect of each extender was evaluated in the presence or absence of seminal plasma at each time point.

**Figure 1:**
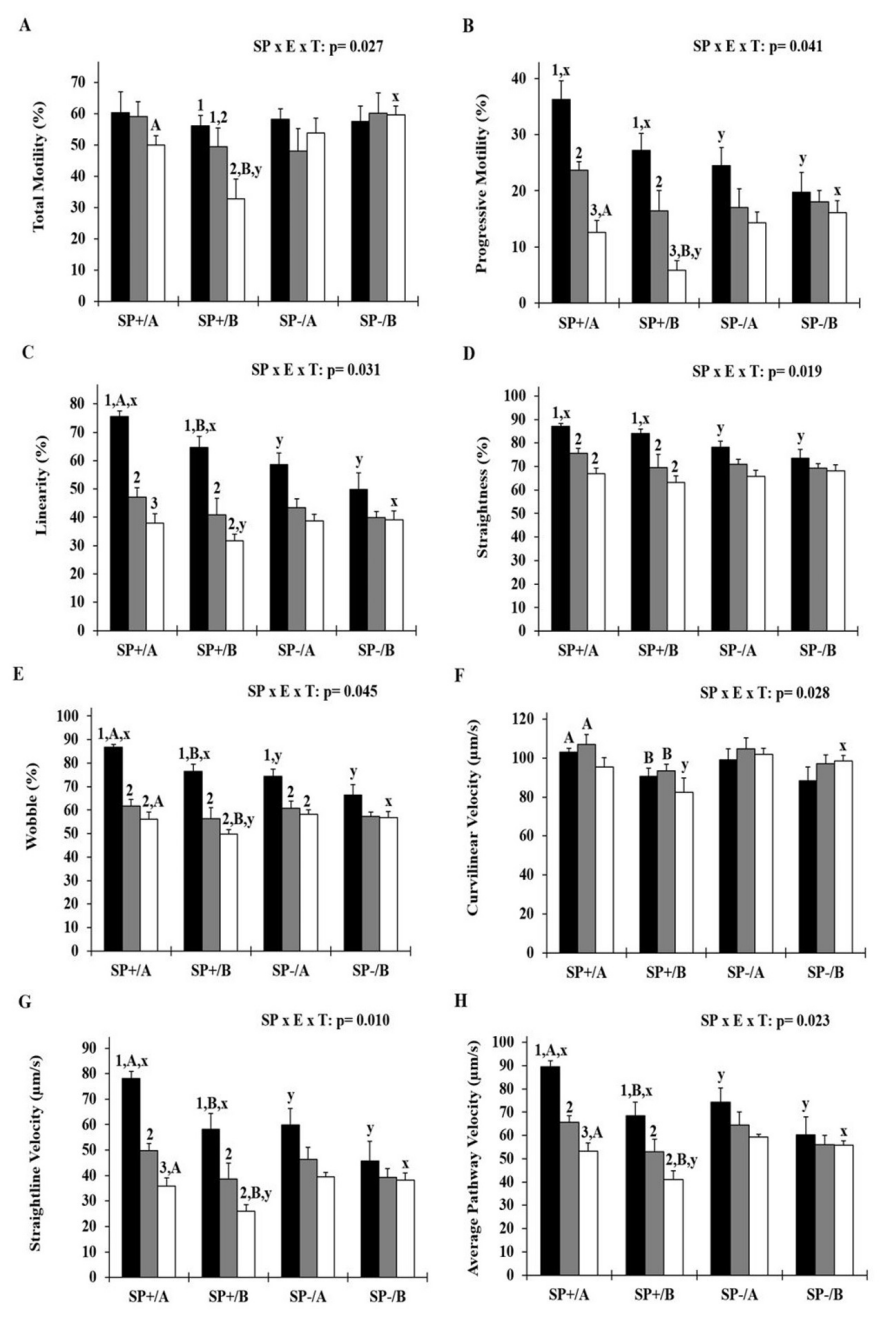
Effect of the removal of seminal plasma and the two soybean lecithin-based extenders on the kinetics of goat sperm cooling for 48 hours.

In samples maintained with seminal plasma (SP+), sperm kinetics (TM, PM, LIN, STR, WOB, VSL and VAP) were reduced after 24 hours of storage ( *p* < 0.05). However, samples cooled in extender A (SP+/A group) showed higher values of TM and PM (at T48), LIN (at T0), WOB, VSL and VAP (at T0 and T48), and VCL (at T0 and T24) than SP+/B group ( *p* < 0.05; Figure 1 AH).

At T0, SP+ groups provided higher values of STR and WOB, regardless of the extender, compared to SP- groups ( *p* < 0.05). On the other hand, at T48, the SP+/B group showed lower values of TM, PM, LIN, WOB, VCL, VSL and VAP compared to SP-/B group ( *p* < 0.05).

**Figure 2:**
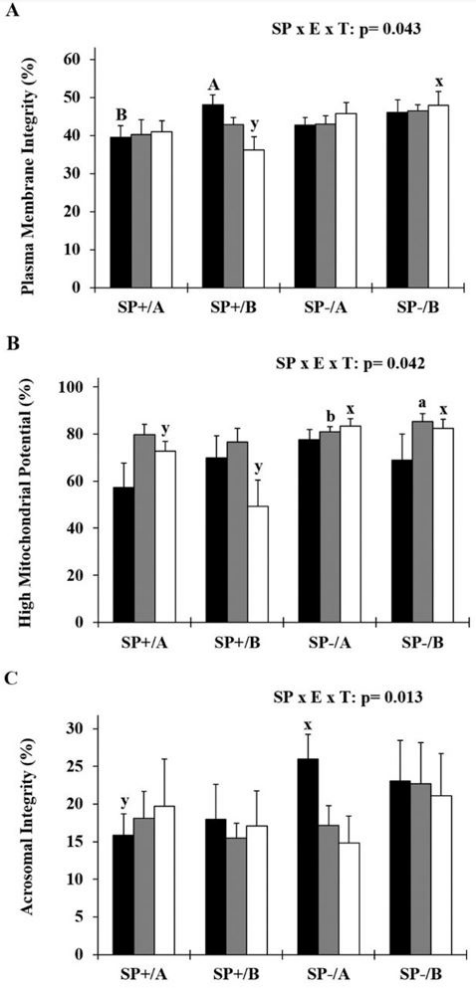
Effect of the removal of seminal plasma and two soybean lecithin-based extenders on the plasma membrane integrity, mitochondrial membrane potential and acrosomal membrane integrity of goat sperm cooling for 48 hours.

In SP+ groups at T0, plasma membrane integrity was higher in extender B compared to extender A ( *p* < 0.01; Figure 2 A). On the other hand, seminal plasma showed deleterious effects on PMI (extender B, T48, *p* < 0.05), HMMP (extender A, *p* < 0.01; and extender B, *p* = 0.016, both at T48) and ACI (extender A, T0, *p* = 0.013), with lower values observed in SP+ samples compared to SP- samples ( Figure 2 AC). Furthermore, in the absence of seminal plasma, HMMP was higher ( *p* < 0.05) in extender B than extender A at T24 ( *p* < 0.05; Figure 2 B).

## Discussion

Our study showed that the removal of goat seminal plasma is a beneficial procedure for the success of the short-term storage of semen. The negative effect of the interaction between goat seminal plasma and SL-phospholipids occurred in a time and concentration-dependent manner.

Buck seminal plasma has lipase enzymes, which have been associated with less resistance to cryopreservation ( Coloma et al., 2010 ); the interaction of the enzymes with the phospholipids of the extenders contributes to the increased rate of semen deterioration ( La Falci et al., 2002 ). In our study, the interaction of seminal plasma with soy lecithin affected mainly sperm kinetics, the values of which decreased significantly after 24 h of refrigeration for samples stored in the presence of seminal plasma.

This deleterious effect of the seminal plasma-extender interaction is related to the level of SL used, since at different times during storage, the sperm kinetics of SP+/A samples (with plasma; 1% SL) were higher than SP+/B samples (with plasma; 2% SL). Therefore, it is possible that the increase of availability of phospholipids in extender B and its constant degradation by seminal lipases contributed to these results. Thus, the hypothesis by Salmani et al. (2014) that the presence of the phospholipase A2 enzyme and goat seminal plasma proteins would require a higher level of SL in the extender does not seem to be confirmed in our study. However, it should be emphasized that Salmani et al. (2014) performed a freeze-thawing process and used 1.5% SL, while in our study 2% SL was used for cooling the goat semen.

In this study, after 48 hours of cooling, the sperm kinetics, plasma membrane integrity and high membrane mitochondrial potential were higher in SP-/B samples (plasma free, 2% SL) compared to SP+/B samples (with seminal plasma, 2% SL). In addition, at T48 the HMMP was also higher in SP-/A (plasma free; 1% SL) than SP+/A (with plasma; 1% SL) groups. These results disagree with the assumption that the goat seminal plasma and SL-phospholipid interactions do not produce toxic effects on sperm ( Roof et al., 2012 ); thus, the results indicate that the recommendation for the removal of seminal plasma through washing/centrifugation in order to increase the success of goat semen freezing, proposed by Ari and Daskin (2010) , should be extended to the chilling of goat semen.

Although the sperm kinetics decreased after 24 h of cooling, it should be noted that the mitochondrial membrane potential was only affected after 48 h of storage, where HMMP was higher in the SP- samples. These results suggest that it is the SL-seminal plasma interaction, and not SL itself, that causes damage to mitochondrial function, as was proposed by Del Valle et al. (2012) and Mata-Campuzano et al. (2015) , who demonstrated that SL may negatively affect the membrane mitochondrial potential of ram sperm. However, due to differences in the species studied and methodologies employed, using the conclusions obtained by Del Valle et al. (2012) and Mata-Campuzano et al. (2015) to explain our results should be carried out with caution, and further studies are needed to confirm whether this mechanism also affects goat spermatozoa.

Our results also suggest that the maintenance of the motility of goat spermatozoa is not totally dependent on ATP produced by mitochondria, as proposed for cattle and sheep ( Martin et al., 2007 ; Del Valle et al., 2012 ); in all groups and evaluations, HMMP was always higher than the number of motile cells, as well as the percentage of cells with intact plasma membranes. In this regard, Qiu et al. (2016) showed that goat sperm can use alternative routes, such as the glycolytic pathway and the phosphate pentose pathway, to maintain motility during storage in the liquid state. Thus, since there was a decrease in motility in the presence of HMMP, it is possible that the loss of plasma membrane integrity, and consequently the leakage of the alternative cytoplasmic pathways, affected the spermatic kinetics. However, further studies should be conducted to elucidate the proposed mechanism.

Therefore, we conclude that the removal of goat seminal plasma is required to improve the quality of goat semen cooling in soybean lecithin-based extender, mainly using 2% soybean lecithin.
